# Type 2-low Asthma: Epidemiology, Multiomics, and Emerging Therapies

**DOI:** 10.1007/s11882-026-01294-1

**Published:** 2026-09-16

**Authors:** Philip Mendez, Alanis Rosado, Juan Carlos Cardet

**Affiliations:** 1 Division of Allergy, Asthma and Immunology, Department of Pediatrics, Macon and Joan Brock Virginia Health Sciences at Old Dominion University, Eastern Virginia Medical School, Norfolk, VA USA; 2 Division of Allergy, Asthma and Immunology, Children’s Hospital of The King’s Daughters, Norfolk, VA USA; 3 Department of Pediatrics, University of South Florida Morsani College of Medicine, Tampa, FL USA; 4 Division of Allergy and Immunology, Department of Internal Medicine, University of South Florida Morsani College of Medicine, Tampa, FL USA

**Keywords:** T2-low asthma, Non-eosinophilic asthma, Neutrophilic asthma, Biomarkers, Non-T2 asthma

## Abstract

**Purpose of Review:**

Type 2-low (T2-low) asthma, characterized by low blood and sputum eosinophils and low fractional exhaled nitric oxide (FeNO), remains a major unmet need because of frequent corticosteroid insensitivity and a lack of targeted therapies. This review summarizes recent advances in its epidemiology, multiomics, and therapeutic landscape.

**Recent Findings:**

T2-low inflammation is highly prevalent, affecting approximately 14%–39% of adults with current asthma and up to 60% of children. Its pathogenesis reflects complex non-T2 mechanisms, including IL-17 signaling, innate immune activation through IL-1β and IL-33, and neutrophilic inflammation. Heterogeneity is further shaped by systemic factors such as obesity and immunosenescence and by environmental exposures such as ozone. Among available therapies, long-term macrolides and the anti-TSLP biologic tezepelumab have shown significant efficacy in reducing exacerbations, whereas other pathway-specific interventions have yielded inconsistent clinical benefits. Emerging metabolic approaches, including GLP-1 receptor agonists, may offer additional promise.

**Summary:**

Progress in T2-low asthma will require mechanism-based classification and clinically deployable biomarkers to align targeted therapies with specific biological drivers.

## Introduction

Asthma is now recognized as a heterogeneous syndrome rather than a single disease, with substantial variability in clinical course, inflammatory pathways, and treatment responsiveness across patients. Contemporary frameworks distinguish “phenotypes” (observable clinical patterns) from “endotypes” (mechanistically defined subgroups), and emphasize biomarker-guided treatment selection to identify patients most likely to benefit from specific therapies [1]. This paradigm shift has been accelerated by the expansion of targeted therapies and by broader clinical use of accessible biomarkers, including blood eosinophil count (BEC), fractional exhaled nitric oxide (FeNO), allergic sensitization and IgE, to guide treatment selection [2]. 

Most progress has centered on type 2 (T2) inflammation, characterized by BEC ≥ 150 cells/µL, sputum eosinophils ≥ 2–3%, and FeNO ≥ 25 ppb [3]. Yet a substantial subset of individuals has T2-low asthma, defined by low blood and sputum eosinophils and FeNO, and frequently demonstrates limited benefit from inhaled corticosteroids (ICS) and modest or absent responses to clinically-available biologics [4–6]. Definitions for this group remain imperfect. Thresholds for BEC and FeNO are debated, T2 biomarkers may be discordant within the same patient, and biomarker levels may vary over time and with treatment within an individual patient (i.e., corticosteroid induced reductions in BEC and FeNO) [7, 8]. 

While T2-high asthma has benefited from a robust therapeutic pipeline and widely accepted biomarkers, diagnostic and therapeutic options for T2-low asthma remain limited. Non-T2 mechanisms likely encompass several overlapping immune mechanisms, including type 1 immunity, driven by interferon gamma (IFN-γ)-associated responses, and type 3 immunity, characterized by IL-17-mediated signaling and neutrophilic airway inflammation [7]. These immune processes may manifest as airway inflammatory phenotypes such as neutrophilic asthma, marked by increased airway neutrophils and corticosteroid insensitivity. Paucigranulocytic asthma is another non-T2 mechanism in which symptoms occur in the absence of prominent inflammatory cell infiltration and may include airway remodeling, airway hypercontractility, fibroblast-associated signaling, and neurogenic or cholinergic dysfunction. In addition, systemic factors, including obesity and metabolic dysfunction, may further modify immune responses and contribute to T2-low disease through inflammatory pathways that do not fit neatly within classical T1 or T3 pathways.

The substantial overlap among these mechanisms underscores the need to move beyond single biomarkers toward multidimensional approaches to asthma phenotyping and treatment. In this review, the term “T2-low asthma” refers to asthma characterized by low levels of clinically available T2 biomarkers (e.g., BEC and FeNO), whereas “non-T2 asthma” refers to the inflammatory and non-inflammatory mechanisms that are not driven by T2 pathways, independent of current biomarker assessments. In this review, the epidemiologic patterns of T2-low asthma across adults and children, pathophysiology, and current and evolving therapeutic strategies are synthesized and explored.

## Epidemiology and Clinical Characteristics

### Adults

Many estimates of the prevalence of T2-low asthma in adults have been based on cohorts of patients with severe or difficult-to-treat asthma and are therefore not representative of broader populations with milder disease. In these enriched cohorts, the reported prevalence varies widely, ranging from approximately 7%–39%, depending on the study population and biomarker definitions [9–16]. For example, in the Greek PHOLLOW cross-sectional study, 20.1% of adults with severe asthma met criteria for T2-low disease [17]. Although prevalence estimates in general adult asthma populations have been scarce, 14.3% of adults with current asthma in the West Sweden Asthma Study met criteria for T2-low asthma, defined by BEC < 150 cells/µL, FeNO < 20 ppb, and absence of clinically allergen-driven disease [18]. Within this cohort, adults with T2-low markers demonstrated distinct demographic characteristics, female sex predominated in the T2-low group (77.3% vs. 59.4% among those with T2-high biomarkers), and median age at asthma onset was later (18.5 vs. 14.0 years), suggesting a tendency toward later-onset disease compared to T2-high asthma.

Smoke exposure does not appear to differ substantially between T2-low and T2-high asthma in population-level analyses [18]. However, mechanistic and cohort studies suggest that cumulative tobacco exposure, air pollution, and occupational irritants may preferentially promote neutrophilic airway inflammation and corticosteroid insensitivity and are characteristic of T2-low subgroups. Aging itself has also been associated with increased airway neutrophilia, potentially contributing to the higher prevalence of T2-low phenotypes in later-onset asthma [19, 20]. 

The clinical burden experienced by patients with T2-low asthma varies by study as well. In the ORACLE study, a meta-analysis of individual-patient data across several randomized controlled trials, elevated T2 inflammatory biomarkers (BEC and FeNO) were both independently associated with higher rates of severe asthma attacks, supporting the concept that exacerbation risk is strongly linked to the presence and magnitude of T2 inflammation [21]. However, population-based studies suggest a more nuanced clinical picture. In a Swedish cohort, individuals with T2-low biomarker profiles had higher rates of asthma-related emergency visits in the preceding year compared to those with high T2 markers, despite better spirometric indices of airflow obstruction [18]. Similar findings have been reported in severe asthma cohorts. The RASP-UK cohort found that T2-low asthma was associated with more asthma-related primary care attendances and a greater likelihood of prior intensive care unit or high-dependency unit admission than T2-high asthma [7]. In the Greek PHOLLOW cohort, a greater proportion of patients with T2-low severe asthma experienced at least one clinically significant exacerbation during the preceding year compared with those with T2-high severe asthma [13]. 

In stable disease, adults with T2-low asthma generally exhibit higher pre-bronchodilator FEV1% predicted and FEV1/FVC ratios than those with T2-high asthma but demonstrate a reduced bronchodilator response [18]. During acute exacerbations, however, physiologic impairment appears similar across phenotypes. In a prospective clinical trial enriched for T2-low severe asthma, exacerbations in T2-low and T2-high disease were associated with a comparable decline in FEV1, FVC, and symptom burden [11]. Notably, these exacerbations were biologically distinct. T2-high exacerbations were characterized by marked increases in FeNO and BEC, whereas T2-low exacerbations showed little to no elevation in T2 biomarkers from baseline. In patients with asthma complicated by bronchiectasis, neutrophilic-dominant exacerbations have been linked to worse in-hospital outcomes, including increased need for intensive care and mechanical ventilation, suggesting that non–T2-driven inflammation confers distinct clinical risk [22]. 

Population-based data likewise show no significant differences in inhaled controller use, ICS dose, or oral corticosteroid use between low- and high-T2 groups [18]. Paradoxically, corticosteroid responsiveness in T2-low asthma is consistently poorer, raising concern for both limited efficacy and increased treatment-related toxicity.

### Pediatrics

As in adults, T2-low asthma has been comparatively understudied in pediatric research. Emerging transcriptomic and population-based data, however, suggest that T2-low asthma may be highly prevalent in children and adolescents.

In a transcriptomic analysis of nasal epithelial samples from three cohorts of Hispanic and African American youths with asthma aged 6 to 20 years, results were classified as T2-high, T17-high, or T2-low/T17-low [23]. Across cohorts, T2-high asthma accounted for 23–29% of participants, whereas T17-high and T2-low/T17-low profiles comprised 35–47% and 30–38%, respectively. These findings challenge the long-standing assumption that pediatric asthma is primarily T2-driven and highlight substantial heterogeneity in airway inflammatory pathways among children and adolescents.

Consistent with this transcriptomic evidence, a nationally representative analysis of U.S. children aged 6–17 years from the 2007–2012 National Health and Nutrition Examination Survey demonstrated a high prevalence of biomarker-defined T2-low asthma. In this cohort, 45.7% of children with current asthma met criteria for T2-low disease, defined as AEC < 300 cells/µL and FeNO < 25 ppb; in a secondary analysis using higher thresholds derived from nasal epithelial transcriptomic profiling (AEC < 428 cells/µL and FeNO < 32 ppb), estimated prevalence approached 60% [24]. 

Across pediatric studies, T2-low asthma commonly shows consistent demographic patterns. Children with T2-low asthma were more likely to have reduced bronchodilator response and be older, female, overweight or obese, exposed to secondhand smoke, and from racial and ethnic minority groups [23, 24]. Together, these findings support the conclusion that T2-low asthma is at least as prevalent as T2-high asthma in pediatric populations and may be substantially underrecognized in routine clinical practice.

These U.S.-based observations occur within the broader context of global asthma epidemiology. Although prevalence patterns vary across regions, asthma remains a leading cause of chronic disease in children worldwide, and environmental exposures–including tobacco smoke, air pollution, and urbanization–continue to shape the distribution and expression of asthma phenotypes across pediatric populations.

## Mechanisms of T2-low Asthma

**Fig. 1:**
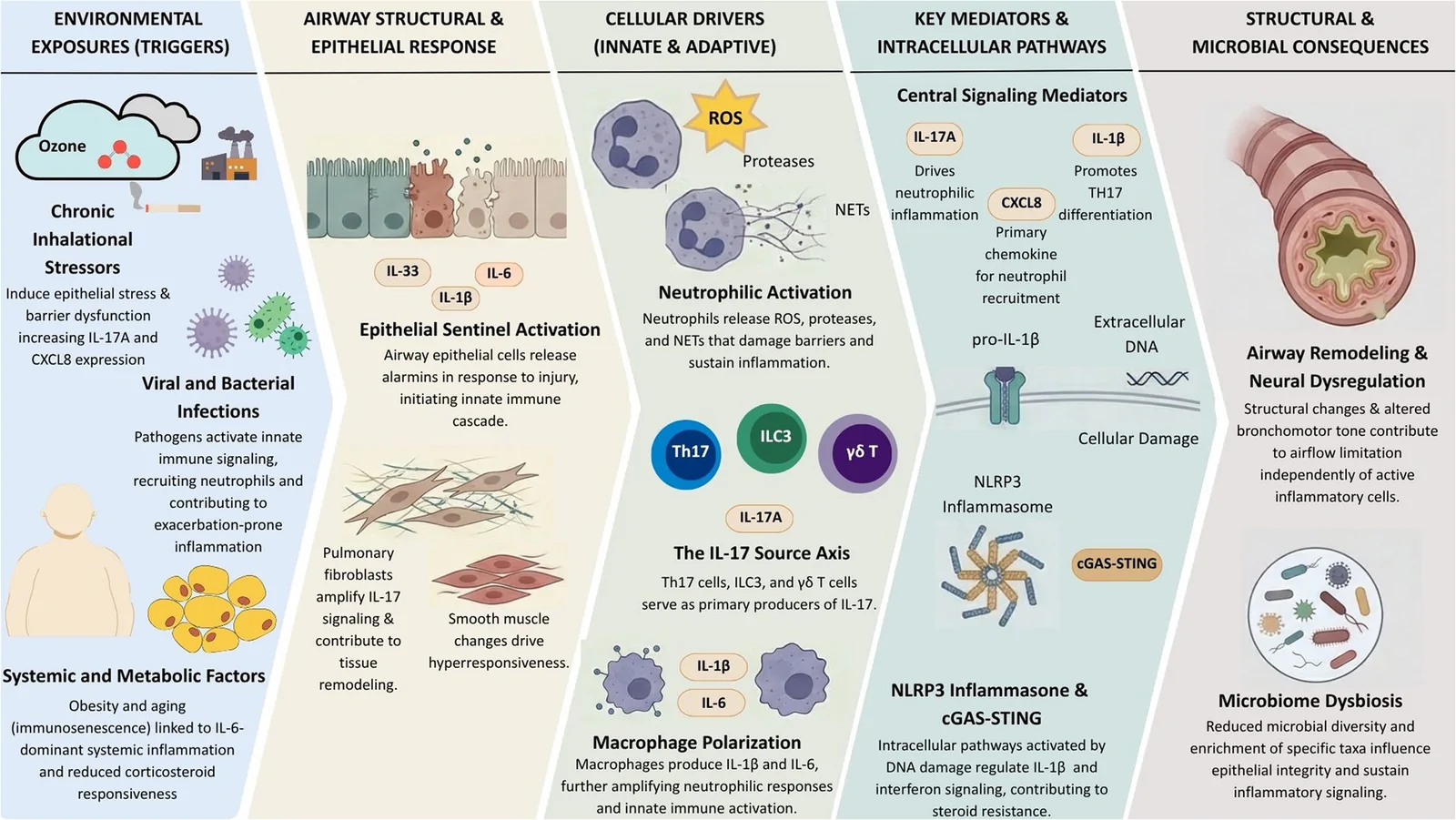
Overview of major mechanisms implicated in T2-low asthma, including environmental and metabolic triggers, epithelial activation, innate and adaptive cellular drivers, key inflammatory mediators and intracellular pathways, and downstream structural and microbial consequences. Prominent features include IL-1β, IL-6, IL-17A, and CXCL8 signaling, neutrophilic inflammation, macrophage and Th17/ILC3/γδ T-cell responses, inflammasome and cGAS-STING pathway activation, airway remodeling, neural dysregulation, and microbiome dysbiosis. ©[sketchify, Eucalyp, Allan Faustino, Slidefactory, Jenzon Lopez, Stelle Butalid, Magtira Paolo] via http://canva.com

 Operational phenotypic definitions in clinical practice rely on biomarker patterns (e.g., BEC, FeNO, allergy tests and total serum IgE) that help distinguish T2-high from non–T2 inflammatory profiles, while also highlighting the instability and overlap of these markers over the natural course of the disease in response to therapies [16, 25, 26]. Rather than reflecting a single inflammatory pathway, T2-low asthma encompasses clinically defined phenotypes arising from overlapping non–type 2 immune processes. These include epithelial barrier dysfunction, innate immune activation involving epithelial-derived signals and myeloid cell responses, neutrophil and macrophage effector responses, IL-17–mediated immunity, airway structural remodeling, systemic metabolic and hormonal influences, microbial–metabolic interactions, and, in some cases, non-inflammatory airway dysfunction (Table 1; Fig. 1) [27].

**Table 1:**
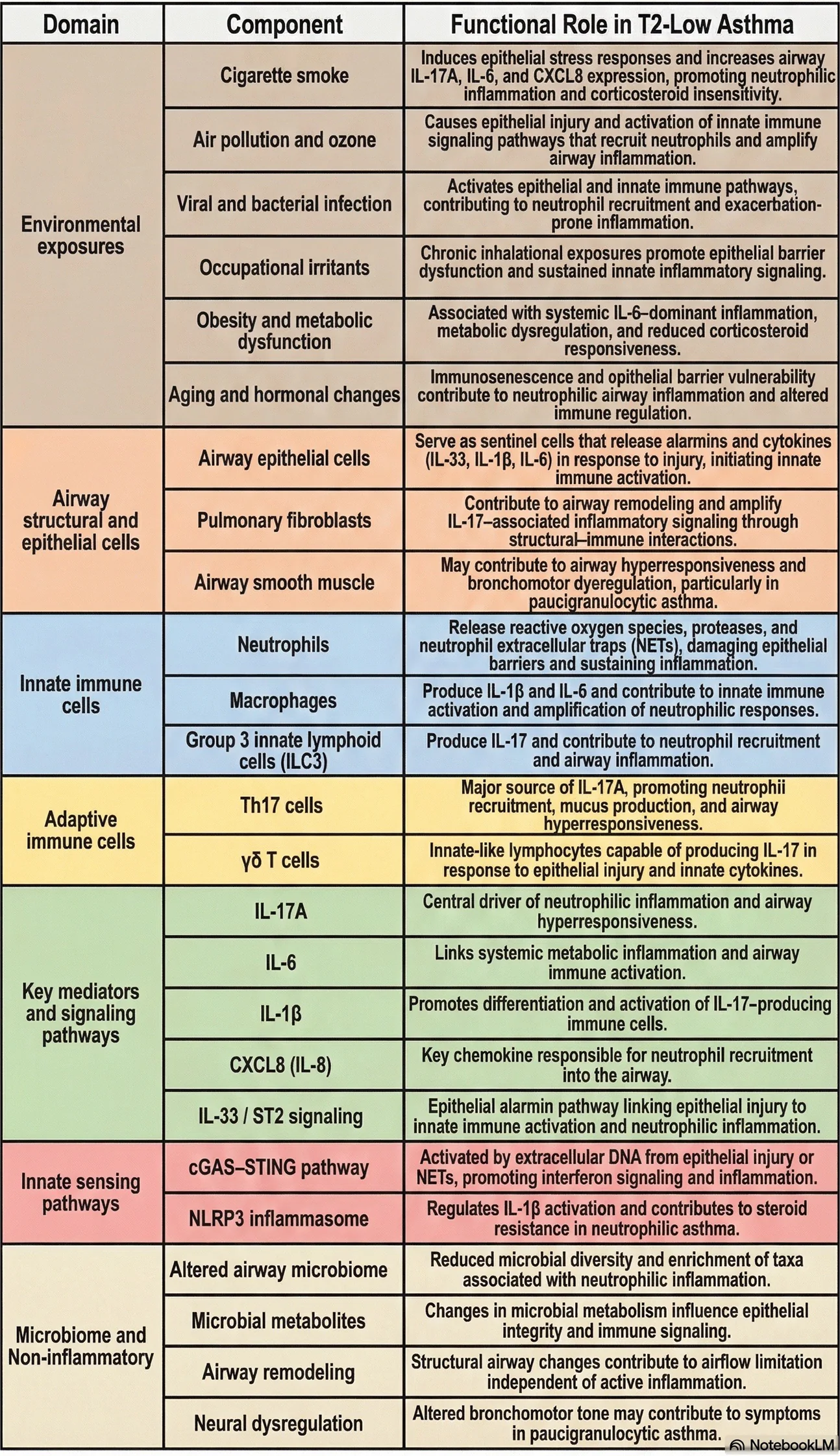
Pathophysiological Drivers and Mechanisms of T2-low Asthma

### Neutrophilic Asthma

Innate immunity contributes to non-T2 airway inflammation through IL-1β, IL-6, and IL-33-associated pathways. IL-1β and IL-6 released from stressed epithelial cells and macrophages promote differentiation and activation of IL-17–producing lymphocytes, while IL-33 may further modulate neutrophilic airway inflammation through ST2, the receptor for IL-33, via downstream signaling in neutrophils, macrophages, and group 3 innate lymphoid cells (ILC3s) [28]. In a translational study by Quoc et al., adults with uncontrolled asthma had elevated serum soluble ST2 and increased ST2 expression on blood neutrophils, with levels inversely correlating with lung function. In vitro, IL-33 enhanced neutrophil activation and led to increases in reactive oxygen species, myeloperoxidase, and NET formation. In a murine model of neutrophilic asthma induced by intranasal sensitization with ovalbumin (OVA) plus lipopolysaccharide (LPS) followed by OVA challenge, anti-ST2 treatment reduced pulmonary neutrophils, macrophages, ILC3s, TNF-α, and IL-17 A [28]. 

Neutrophils contribute to airway pathology in non-T2 asthma through degranulation, release of reactive oxygen species, and formation of neutrophil extracellular traps (NETs), which damage epithelial barriers and perpetuate inflammation [29]. In pediatric asthma, sputum myeloperoxidase and neutrophil gelatinase-associated lipocalin correlate with airflow limitation and symptom burden even when neutrophil counts are not markedly elevated, suggesting that neutrophil activation state rather than absolute cell number may be what drives clinical impact [15]. 

Innate sensing by epithelial cells of extracellular host nucleic acids and other injury-associated ligands amplifies neutrophilic inflammation. Extracellular double-stranded DNA released from dying epithelial cells and NETs activates the cGAS–STING pathway, inducing type I interferons, IL-6, CXCL10, and epithelial PANoptosis, which is a coordinated inflammatory cell death program integrating pyroptotic (gasdermin-mediated lytic inflammatory death), apoptotic (caspase-mediated programmed cell death), and necroptotic pathways (RIPK-dependent lytic programmed necrosis) [30]. In murine models, STING agonism during allergen exposure induces severe neutrophilic inflammation, barrier disruption, NET formation, and mixed Th1/pro-inflammatory cytokine responses that recapitulate features of severe asthma. NETosis itself is increasingly recognized as a central pathogenic mechanism: transcriptomic analysis identifies NET-related gene programs as dominant in neutrophilic asthma, with NCF1 emerging as a key regulator associated with macrophage–neutrophil interactions, airway remodeling, and glucocorticoid resistance [31]. NET-derived damage-associated molecular patterns further amplify inflammation through TLR4/NF-κB signaling and epithelial injury, establishing a feed-forward loop that sustains neutrophilic disease. In parallel, expanded biomarker frameworks for extracellular traps have been proposed (spanning neutrophil, monocyte, and macrophage-derived traps), reflecting increasing interest in distinguishing NET-dominant biology from neutrophilia defined only by cell counts [29, 32–34]. 

IL-17 A contributes to non-T2 airway inflammation by promoting neutrophil recruitment, mucus hypersecretion, and airway hyperresponsiveness and can be produced by Th17 cells, γδ T cells, and ILC3s in response to IL-1β and IL-23 [35]. Pulmonary fibroblasts may further amplify this pathway through stem cell factor (SCF)–c-Kit signaling, particularly at the level of ILC3s. In a recent translational study, SCF enhanced IL-1β/IL-23-driven ILC3 expansion and IL-17 A production, and complementary murine models showed that genetic or pharmacologic interruption of this pathway attenuated neutrophilic inflammation and airway hyperresponsiveness.

Cadherin-11 (CDH11), a mesenchymal adhesion molecule upregulated in bronchial mucosa in severe asthma, has also been implicated in neutrophilic airway inflammation. In a murine model, CDH11 antagonism reduced airway neutrophilia and IL-6/TNF-α production [36]. Recombinant CDH11 also induced neutrophil recruitment, which was attenuated by FGFR1 inhibition, supporting a structural–immune interaction partly mediated through FGFR1 signaling.

### Paucigranulocytic Asthma

Paucigranulocytic asthma, defined by the absence of both eosinophilic and neutrophilic airway inflammation, represents a substantial subset of T2-low asthma, particularly among treated and clinically stable patients [14]. Symptoms in this group may reflect airway smooth muscle hypercontractility, altered neural regulation of bronchomotor tone, epithelial dysfunction, or airway remodeling rather than active inflammation, underscoring that T2-low asthma is not synonymous with neutrophilia and that non-inflammatory mechanisms may dominate in some patients.

Neural dysfunction may contribute to airway hyperresponsiveness in a subset of paucigranulocytic asthma, although direct evidence in this phenotype remains limited. In a murine study, MrgprC11-positive vagal sensory neurons comprised only about 2% of total vagal sensory neurons, yet their activation triggered reflex cholinergic bronchoconstriction and increased airway responsiveness even without overt inflammation, supporting a potential role for neurogenic mechanisms independent of granulocytic airway infiltrates [37]. More broadly, airway sensory and parasympathetic nerves regulate bronchomotor tone through reflex pathways in which acetylcholine-mediated M3 receptor activation promotes bronchoconstriction, whereas inhibitory neuronal M2 receptors restrain excessive acetylcholine release [38, 39]. At the same time, sputum profiling in adolescents and young adults with asthma found no clear increase in neural mediators in noneosinophilic asthma, suggesting that neural dysfunction is biologically plausible in pauci-inflammatory disease but not yet established as a dominant mechanism in this phenotype [40]. 

One potential mechanistic contributor is the 17q21 locus, particularly ORMDL3-associated biology linked to childhood-onset asthma and airway hyperreactivity. ORMDL3 regulates sphingolipid biosynthesis and has been implicated in epithelial dysfunction, airway remodeling, and nonallergic asthma-related pathways. Experimental studies show that increased ORMDL3 expression in airway smooth muscle enhances proliferation, contractility, and calcium oscillations and can promote airway hyperresponsiveness even in the absence of airway inflammation, partly through SERCA2b-associated calcium handling [41]. Complementing this, children with 17q21 risk genotypes show reduced de novo sphingolipid synthesis, and those with low-eosinophil asthma have lower whole-blood dihydroceramides, ceramides, and sphingomyelins than controls, further supporting a noncanonical metabolic-structural pathway to airway dysfunction [42]. 

Another mechanistic framework centers on G protein-coupled receptor signaling and its regulation by RGS proteins, which modulate bronchoconstrictive airway smooth muscle responses. Reduced RGS2 expression augments Gαq-mediated calcium signaling in human airway smooth muscle, and in SARP-3, risk-allele homozygotes for rs2746071/rs2746072 had nearly twofold higher exacerbation rates in non-Hispanic White patients, linking impaired signal termination to a clinically relevant hypercontractile phenotype [43]. Experimental repression of RGS2 also worsens airway hyperresponsiveness and remodeling, including peribronchial smooth muscle thickening and fibrosis, in murine models [44]. In parallel, RGS4 influences airway responsiveness through both GPCR-dependent and GPCR-independent mechanisms; GAP-deficient RGS4 knock-in mice had increased methacholine responsiveness and reduced airway PGE2, implicating abnormal epithelial bronchodilator counter-regulation as another possible contributor to pauci-inflammatory airway dysfunction [45]. 

### Microbiome, Obesity, and Aging in Non-T2 Asthma

Airway microbiome alterations are not unique to non-T2 asthma. In a study of steroid-naive atopic asthma, Durack et al. defined T2-high disease using a bronchial epithelial three-gene mean (TGM) expression score derived from CLCA1, SERPINB2, and POSTN. A TGM score of ≥ 1.117, corresponding to two standard deviations above the mean score in healthy controls, was used to define T2-high asthma; 10 of 40 asthmatic participants met criteria for T2-high asthma [46]. TGM scores correlated positively with serum IgE (rS = 0.36, *p* < 0.01) and blood and sputum eosinophils (rS = 0.32 and 0.37, respectively; *p* ≤ 0.01), and inversely with FEV1 (rS = − 0.45, *p* < 0.005) and methacholine PC20 (rS = − 0.41, *p* < 0.005). Subjects with T2-high asthma had significantly lower bronchial bacterial burden than those with T2-low asthma (*p* = 0.04), and bacterial burden correlated inversely with TGM scores across asthmatic participants (rS = − 0.43, *p* < 0.01), independent of age (*p* = 0.03). However, because only 4 T2-high samples yielded sufficient sequencing material for profiling, compositional differences between T2-high and T2-low asthma could not be robustly assessed. More specifically, other studies have linked neutrophilic asthma or sputum neutrophilia to reduced microbial diversity, relative dominance of taxa such as *Haemophilus influenzae*, and enrichment of neutrophil-associated host-response pathways, including NETosis and IL-6 trans-signaling [47–53]. Integrated metabolomic analyses have identified distinct pathway-level signatures across asthma phenotypes rather than a uniform increase in microbial-derived metabolites. In one sputum multi-omics study, neutrophilic asthma versus healthy controls was characterized primarily by enrichment of tryptophan metabolism, with quinolinic acid, 5-hydroxyindole-3-acetic acid, acetyl-N-formyl-5-methoxykynurenamine, and 2-ketoadipic acid emerging as top discriminatory metabolites; eosinophilic asthma versus healthy controls was associated with pyrimidine metabolism, whereas eosinophilic versus neutrophilic asthma was distinguished most strongly by arachidonic acid metabolism [47]. These phenotype-specific metabolomic differences were linked to distinct airway microbial patterns, supporting a potential role for microbiome–metabolite interactions in shaping local inflammatory programs.

Obesity-associated inflammation and age-related hormonal shifts skew airway immunity toward non-T2 inflammatory pathways. Plasma IL-6 correlates with obesity, insulin resistance, and asthma severity independent of eosinophilic inflammation, and genetic analyses demonstrate shared loci between obesity-related traits and later-onset, non-atopic asthma [54–56]. 

Aging introduces a distinct immunologic context in which neutrophilic and T2-low asthma are more likely to emerge. Immunosenescence, the age-related deterioration of immune function, is characterized by impaired mucociliary clearance, epithelial barrier vulnerability, altered antigen presentation, and chronic low-grade systemic inflammation [57]. In older adults, bronchoalveolar lavage and sputum studies demonstrate increased airway neutrophils even in the absence of overt disease, and in elderly asthmatics, sputum neutrophilia is associated with lower FEV₁ and air trapping [58]. Menopause-related immune remodeling adds another layer of heterogeneity in older women, with estrogen deprivation associated with increased circulating pro-inflammatory cytokines, including IL-1, IL-6, and TNF-α, along with reductions in CD4 + T and B lymphocyte counts and natural killer (NK) cell function; these changes may plausibly contribute to late-onset, non-atopic and steroid-resistant patterns observed in clinical practice [59]. This heterogeneity is reflected in recent clinical phenotyping data showing that neither airway nor blood neutrophilia alone defines a stable clinical phenotype. In the ATLANTIS cohort, sputum and blood neutrophil counts were not independently associated with symptom burden, decreased lung function, or exacerbation risk, underscoring that neutrophilic inflammation must be interpreted in biological and clinical context rather than as a stand-alone disease marker [60]. 

### Environmental and Exogenous Factors

Environmental exposures such as cigarette smoke, air pollution, and microbial products may contribute to T2-low epithelial stress responses and innate immune activation that differ from allergen-driven type-2 pathways [16]. Cigarette smoke is best viewed not as a defining marker of T2-low asthma, but as an exposure that can promote non-eosinophilic disease features, including airway neutrophilia, IL-17/IL-6/IL-8-associated inflammation, and reduced corticosteroid responsiveness, while still overlapping biologically with eosinophilic disease in some patients [61–65]. 

Ozone may promote T2-low airway disease by activating innate inflammatory pathways that favor neutrophilic inflammation, including IL-1β/IL-17 A signaling and, in experimental models, IL-6/STAT3-associated corticosteroid resistance [66–68]. In controlled human exposure studies, 6.6 h of sedentary exposure to ozone at an average concentration of 70 ppb caused a significant decline in percent-predicted FEV1 and increased sputum neutrophils, supporting clinically relevant proasthmatic effects even at currently relevant exposure levels [66]. Among African American adolescents with persistent asthma receiving guideline-based controller therapy, short-term increases in ambient ozone below the National Ambient Air Quality Standard were likewise associated with lower percent-predicted FVC and FEV1, suggesting persistent susceptibility despite treatment [69]. 

Rhinovirus may represent an additional viral trigger relevant to this inflammatory landscape. In differentiated human bronchial epithelial cells, rhinovirus induced IL-1α and the neutrophil-attractant cytokines IL-6, IL-8, and CXCL10, while IL-1 receptor antagonism attenuated these responses without significantly changing interferon-β release or viral titer [70]. In complementary experimental models, rhinovirus-triggered exacerbation was associated with an early neutrophilic phase characterized by IL-1α, IL-1β, CXCL1, and NETosis, and this response was abrogated by blockade of IL-33 release/signaling [71]. Additional murine data further implicate a CXCL3/CXCL5/CXCR2 chemotactic axis in rhinovirus-induced exacerbation, as inhibition of CXCL3 or CXCL5 reduced CXCR2 + neutrophil accumulation, airway hyperreactivity, mucus hypersecretion, and type 2 regulatory mediators [72]. 

Taken together, T2-low asthma represents a spectrum of overlapping but distinct pathophysiologic processes involving epithelial injury, innate immune activation, IL-17-driven immunity, neutrophil and macrophage effector pathways, structural cell signaling, microbial–metabolic modulation, systemic metabolic and hormonal influences, and, in some cases, non-inflammatory airway dysfunction. This mechanistic heterogeneity explains the limited efficacy of uniform treatment strategies and highlights the need for multidimensional phenotyping and targeted therapeutics beyond classical type-2 pathways.

## Current and Emerging Therapeutic Approaches in T2-Low Asthma

The management of T2-low asthma remains challenging due to limited responsiveness to ICS, heterogeneity of underlying mechanisms, and the absence of validated biomarkers to guide treatment selection. Current treatment guidelines do not distinguish inhaled controller selection on the basis of T2-high versus T2-low status alone. Unlike T2-high asthma, where downstream cytokine blockade yields predictable benefit, therapeutic strategies for T2-low asthma increasingly emphasize targeting upstream epithelial signaling and non-T2 pathways, as well as identification of treatable traits rather than strict inflammatory endotypes [73–75]. 

### Macrolide Therapy for Neutrophilic and Non-eosinophilic Asthma

Long-term macrolide therapy represents the most robust non-biologic intervention for T2-low asthma to date. Azithromycin exerts immunomodulatory effects independent of antimicrobial activity, including suppression of IL-8 and IL-17 signaling, inhibition of neutrophil chemotaxis, enhancement of antiviral responses, and modulation of airway microbial communities [74]. The mechanism most responsible for its clinical benefit remains unclear. Post hoc and microbiome data suggest that reduction of airway bacterial burden, particularly *Haemophilus influenzae*, may contribute importantly to benefit, but this has not been definitively established.

The AMAZES randomized controlled trial evaluated azithromycin 500 mg three times weekly for 48 weeks in adults with persistent uncontrolled asthma despite ICS-based therapy. In the overall cohort, azithromycin reduced the annualized exacerbation rate from 1.86 to 1.07 events per patient-year, corresponding to an incidence rate ratio (IRR) of 0.59 (95% CI 0.47–0.74; *p* < 0.0001). Importantly for T2-low asthma, prespecified subgroup analyses demonstrated comparable benefit in non-eosinophilic asthma (BEC < 200 cells/µL), with exacerbation rates reduced from 1.98 to 1.09 events per year (IRR 0.55; 95% CI 0.36–0.85; *p* = 0.007). Asthma-related quality of life scores improved by a mean of 0.36 points on the AQLQ (95% CI 0.21–0.52; *p* < 0.001), exceeding the minimal clinically important difference, while pre-bronchodilator FEV₁ changes were modest and not statistically significant [76]. 

These findings are reinforced by multiple meta-analyses. A 2023 ERS meta-analysis of long-term macrolide therapy (≥ 24 weeks) encompassing over 1,500 participants reported a pooled reduction in exacerbation frequency with a rate ratio of 0.62 (95% CI 0.50–0.77), with greater relative benefit observed in studies enriched for non-eosinophilic or neutrophilic phenotypes [77]. Sputum neutrophil percentages decreased by a weighted mean difference of approximately − 7% to − 10% across studies, although heterogeneity was substantial and correlations with lung function were inconsistent. A Cochrane review similarly found reduced exacerbations (OR 0.64; 95% CI 0.50–0.82) but no consistent improvement in FEV₁, highlighting the dissociation between symptom control and spirometric outcomes in neutrophilic asthma [78]. Consistent with these data, GINA includes low-dose azithromycin as an add-on option for adults with severe asthma, and the most recent ERS/ATS severe asthma guideline suggests a trial of macrolide therapy in persistently symptomatic or uncontrolled adults at Step 5 [3, 79]. However, risks related to antimicrobial resistance, QT prolongation, and microbiome disruption necessitate careful patient selection and longitudinal monitoring.

Despite robust evidence supporting azithromycin as add-on maintenance therapy for adults with uncontrolled or severe asthma, data supporting maintenance azithromycin in pediatric asthma remains limited, particularly for biomarker-defined T2-low disease. In an open-label randomized trial of children with poorly controlled asthma, adjunctive azithromycin administered three times weekly for 3 months improved asthma-control scores and reduced exacerbations, with similar clinical benefits observed in post-hoc eosinophilic and noneosinophilic subgroups. However, azithromycin did not significantly improve spirometry measures, including FEV1, FVC, FEV1/FVC, and peak expiratory flow, or measures of airway inflammation, including FeNO and sputum neutrophil percentages [80]. An earlier placebo-controlled study of only 16 children reported reductions in bronchial hyperresponsiveness and sputum neutrophils after 8 weeks of treatment without improvement in baseline lung function [81], whereas a multicenter ICS-sparing trial was terminated early for futility after finding no benefit of azithromycin in maintaining control during ICS reduction [82]. These findings support the need for further pediatric trials evaluating long-term azithromycin in children with T2-low asthma.

### Targeting Epithelial Alarmins

Epithelial alarmins represent a central therapeutic target in T2-low asthma by integrating environmental injury, infection, and metabolic stress into downstream immune activation through non-T2 inflammatory pathways [83]. Thymic stromal lymphopoietin (TSLP) and interleukin-33 (IL-33) activate innate and adaptive immune pathways that extend beyond classical T2 inflammation, including Th1, Th17, neutrophil, and macrophage responses.

Tezepelumab, a monoclonal antibody targeting TSLP, is currently the only approved biologic demonstrating efficacy across the T2 phenotypic spectrum. In pooled analyses of the PATHWAY and NAVIGATOR trials, tezepelumab reduced annualized exacerbation rates by 60% compared with placebo (rate ratio 0.40; 95% CI 0.34–0.48). Importantly, efficacy extended to patients with T2-low asthma: patients with baseline BEC < 300 cells/µL experienced a 41% reduction in exacerbations (rate ratio 0.59; 95% CI 0.46–0.75), while those with FeNO < 25 ppb had a 39% reduction (rate ratio 0.61; 95% CI 0.48–0.78) [84]. 

Improvements in lung function were modest but statistically significant overall (mean pre-bronchodilator FEV₁ increase ~ 130 mL vs. placebo; *p* < 0.001), with smaller and more variable gains in T2-low subgroups [84]. Biomarker analyses showed reductions in BEC, FeNO, and IgE, underscoring TSLP’s role as an upstream inflammatory regulator even in patients with T2-low asthma.

Real-world evidence from the PASSAGE phase 4 study demonstrated a 76% reduction in exacerbations (95% CI 69–81) across phenotypes, including patients with low eosinophils, smoking history, and comorbid COPD, with mean FEV₁ improvement of 0.11 L at 24 weeks (95% CI 0.06–0.17). Smaller real-world cohorts similarly report improved asthma control and reduced oral corticosteroid use irrespective of T2 status [85, 86]. 

IL-33 blockade represents a parallel alarmin strategy with evolving evidence. Astegolimab (anti-ST2) reduced exacerbations by 43% versus placebo in a phase 2b trial (ZENYATTA), with numerically greater effects in patients with low eosinophil counts, although subgroup analyses were underpowered [87]. Itepekimab (anti-IL-33) reduced loss-of-asthma-control events from 41% to 22% overall, with a favorable but non-definitive signal in patients with baseline eosinophils < 300 cells/mm³ (19% vs. 33%) [88]. However, not all mechanistically relevant non-T2 pathways have translated successfully into effective targeted therapies. For example, brodalumab (anti-IL-17RA) showed no overall benefit in moderate-to-severe asthma, and risankizumab (anti-IL-23) was not beneficial in severe asthma, underscoring the ongoing challenge of identifying which components of non-T2 inflammation are therapeutically actionable [89, 90]. 

Mechanistic studies caution that IL-33 may exert context-dependent regulatory and tissue-repair functions in models of neutrophilic and irritant-induced airway inflammation. In experimental models characterized by non-allergic, neutrophil-predominant disease, IL-33 expression was induced following epithelial injury and contributes to attenuation of airway injury, rather than amplification of inflammation; disruption of IL-33 signaling in these settings has been associated with worsened airway hyperresponsiveness or impaired recovery after inflammatory insult [91, 92]. In parallel, mechanistic studies of alarmin biology demonstrate that IL-33/ST2 signaling promotes epithelial repair and immune homeostasis, including induction of amphiregulin and expansion of ST2-expressing regulatory T cells, supporting restoration of airway barrier integrity after injury [93, 94]. Consistent with this complexity, combined IL-33 and IL-4Rα antagonism did not outperform either monotherapy in a phase 2 asthma trial [88]. Collectively, these findings suggest that while IL-33 blockade may reduce exacerbation risk in selected patients, its dual role in inflammation and repair may limit the benefit of indiscriminate inhibition in certain T2-low or neutrophilic asthma endotypes, underscoring the need for refined endotype-specific patient selection.

### Targeting Neutrophil Recruitment and Chemokine Signaling

Neutrophilic inflammation in T2-low asthma is sustained by chemokine networks centered on CXCL8 and its receptor CXCR2. Pharmacologic inhibition of CXCR2 reduces neutrophil recruitment and activation in preclinical models and early-phase clinical trials, but clinical benefits were modest, with minimal or inconsistent improvements in asthma control, lung function, and exacerbation outcomes [95]. In a larger phase 2b trial, AZD5069 failed to reduce severe exacerbations or improve lung function and symptom control despite dose-dependent reductions in circulating neutrophils [96]. Together, these findings suggest that CXCR2-mediated neutrophil trafficking alone is unlikely to be a sufficient therapeutic target in unselected T2-low asthma and highlight the need for more precise biomarker-defined approaches.

### Metabolic Modulation

Obesity-associated asthma, a common T2-low phenotype, is characterized by IL-6–dominant inflammation, metabolic dysfunction, and reduced corticosteroid responsiveness [74]. Emerging preclinical data demonstrate that glucagon-like peptide-1 receptor signaling suppresses epithelial IL-33 release and downstream innate lymphoid activation, raising the possibility that metabolic therapies may indirectly modulate upstream inflammatory drivers in patients with T2-low asthma [97]. This concept is now being tested clinically in the ongoing GATA-3 trial, a randomized, placebo-controlled phase 2 proof-of-concept study of semaglutide in adults with symptomatic asthma despite ICS therapy and excess body weight/obesity [98, 99]. The trial is designed to evaluate whether semaglutide improves asthma control and reduces airway inflammation, reflecting growing interest in metabolically targeted approaches for obesity-associated asthma. Although human data remain limited, these findings align with a treatable-traits framework integrating metabolic, inflammatory, and airway-structural contributors to disease.

## Future Steps

Advancing therapies for non-T2 asthma requires moving beyond definitions based on the *absence* of type 2 biomarkers and moving toward those based on the presence of mechanism-informed biomarkers that can be identified and targeted in routine clinical practice. Non-T2 asthma comprises multiple biologically distinct processes, and commonly used biomarkers may be discordant, treatment-sensitive, and temporally variable. Future progress therefore depends on better disease stratification, clinically deployable biomarkers, and trial designs that reflect phenotypic overlap and evolution.

Longitudinal phenotyping should be incorporated into cohort studies and clinical trials. Phenotype overlap and evolution across the lifespan suggest that a single baseline biomarker assessment may misclassify patients, particularly those receiving corticosteroids or sampled only during clinical stability. Serial assessment is particularly important because non–T2 inflammatory activity may vary with seasonal respiratory infections, air pollution, and environmental irritant exposures rather than allergen-driven mechanisms. Such approaches may clarify transitions between T2-high, T2-low, and mixed inflammatory phenotypes.

Multiomics strategies are likely to outperform single-threshold approaches. Current T2 biomarkers do not consistently predict biologic response, supporting composite models that integrate transcriptomic, proteomic, and metabolomic data with systemic inflammatory markers, metabolic traits, environmental exposures, and indicators of epithelial injury or neutrophil activation. However, data-driven clusters require mechanistic validation to ensure correspondence with actionable biological pathways.

Pediatric and life-course trajectories require dedicated investigation. Pediatric asthma clusters differ substantially from adult disease, and endotype definitions cannot simply be extrapolated across age groups. Important work from longitudinal birth and childhood cohort studies has already provided a strong foundation for understanding asthma trajectories across the lifespan. Building on this framework, future studies should evaluate how early-life exposures—including viral infections, secondhand smoke, environmental pollutants, and metabolic risk factors—interact with airway immune development and structural remodeling. Determining whether early non–T2-high signatures predict disease persistence, airway remodeling, or transition to mixed inflammatory phenotypes may inform preventive and early targeted strategies. Broader asthma-burden data also suggest that healthcare access, obesity, tobacco exposure, and environmental context may influence the expression and consequences of non–type 2 disease, underscoring the need for more geographically diverse, phenotype-specific studies [100, 101]. 

Therapeutic development should align targets with biologically enriched populations. Clinical trials should enroll mechanistically defined subgroups (IL-17–dominant disease, neutrophil-activation signatures, metabolic or systemic inflammatory phenotypes, or paucigranulocytic airway dysfunction) rather than broad “non-eosinophilic” populations. Multiomics-derived endotypes may be particularly useful for identifying such subgroups and matching them to relevant pathways. Upstream epithelial–innate pathways represent attractive targets but their effects may differ across neutrophilic versus paucigranulocytic mechanisms and should be evaluated accordingly.

Clinical outcomes should extend beyond conventional spirometric measures. Relevant endpoints include acute exacerbation biology, corticosteroid exposure and toxicity, patient-reported outcomes, healthcare utilization, and comorbidity-centered measures such as metabolic dysfunction or infection susceptibility. Progress will also require phenotyping acute exacerbations themselves, as emerging evidence suggests that asthma attacks differ in their inflammatory drivers, biomarker profiles, microbial associations, and corticosteroid responsiveness. Integrating biomarker-informed assessment during exacerbations may therefore help identify biologically distinct attack subtypes and support more precise acute management strategies [102]. 

Ultimately, progress in T2-low asthma will require integration of mechanistic insights into clinical decision-making. Dynamic models incorporating longitudinal biomarkers, environmental exposures, treatment responses, and multiomics data may enable personalized risk stratification and therapy approaches. As epidemiologic and mechanistic evidence evolves, T2-low asthma is increasingly recognized as a spectrum of overlapping inflammatory and non-inflammatory processes shaped by environmental exposures, metabolism, aging, and airway structural changes. Advancing care will therefore depend on transitioning from static, exclusion-based definitions toward pathophysiology-anchored frameworks that support precision medicine.

## Key References

- Porpodis K, Zias N, Kostikas K, Tzouvelekis A, Makris M, Konstantinou GN, et al. T2-low severe asthma clinical spectrum and impact: The Greek PHOLLOW cross‐sectional study. Clin Transl Allergy. 2025 Feb;15(2):e70035. https://doi.org/10.1002/clt2.70035.
  - ○ This study provides real-world evidence that T2-low asthma constitutes a substantial proportion of severe asthma in specialty care settings. It helps define the clinical relevance of this phenotype beyond controlled trial populations and underscores the scale of the unmet therapeutic need.
- Ercan S, Abohalaka R, Ilmarinen P, Lehtimäki L, Ermis SSO, Lisik D, et al. Characteristics of Adult Asthma Based on Type 2 Inflammation Markers: A Population-Based Study. J Allergy Clin Immunol Pract. 2025 Nov;13(11):2970–2979.e6. https://doi.org/10.1016/j.jaip.2025.07.009.
  - ○ This study is important because it moves the adult T2-low literature beyond enriched severe-asthma cohorts and provides a population-based estimate of prevalence. It also reinforces the idea that low T2 biomarker status does not equate to low clinical burden, which has implications for risk stratification in routine practice.
- Yue M, Gaietto K, Han YY, Rosser FJ, Xu Z, Qoyawayma C, et al. Transcriptomic Profiles in Nasal Epithelium and Asthma Endotypes in Youth. JAMA. 2025 Jan 28;333(4):307. https://doi.org/10.1001/jama.2024.22684.
  - ○ This study represents a major advance in pediatric asthma endotyping by demonstrating that transcriptomic profiles can identify biologically distinct T2-high, T17-high, and non–T2-high groups in youth. It challenges reliance on peripheral biomarkers alone and supports a shift toward airway-based molecular classification early in the disease course.
- Han YY, Gaietto K, Yue M, Rosser FJ, Chen W, Celedón JC. Prevalence and Potential Risk Factors for T2-Low Asthma Among School-Aged Children in the National Health and Nutrition Examination Survey, 2007–2012. J Allergy Clin Immunol Pract. 2025 Aug;13(8):2075–2082.e2.https://doi.org/10.1016/j.jaip.2025.04.040.
  - ○ This paper provides nationally representative U.S. pediatric data and directly supports one of the review’s central epidemiologic messages. The finding that roughly half of school-aged children with current asthma met criteria for T2-low disease challenges the longstanding assumption that childhood asthma is predominantly T2-high.
- Quoc QL, Cao TBT, Jang JH, Shin YS, Choi Y, Park HS. ST2-Mediated Neutrophilic Airway Inflammation: A Therapeutic Target for Patients With Uncontrolled Asthma. Allergy Asthma Immunol Res. 2024;16(1):22.https://doi.org/10.4168/aair.2024.16.1.22.
  - ○ This study helps bridge observational and mechanistic work on the IL-33/ST2 axis by linking biomarker abnormalities in uncontrolled asthma to functional effects on neutrophils and macrophages. Its importance lies in strengthening the rationale for IL-33/ST2 as a plausible non-T2 therapeutic target, while also illustrating how biomarker-defined neutrophilic disease may be tied to specific innate immune pathways rather than neutrophilia alone.
- Versi A, Azim A, Ivan FX, Abdel-Aziz MI, Bates S, Riley J, et al. A severe asthma phenotype of excessive airway *Haemophilus influenzae* relative abundance associated with sputum neutrophilia. Clin Transl Med. 2024 Sep;14(9):e70007. https://doi.org/10.1002/ctm2.70007.
  - ○ This paper is important because it advances the concept that at least some non-T2 asthma subgroups may be microbiologically defined rather than identified solely by inflammatory cell counts. By linking a specific airway organism to a neutrophilic host-response program, it supports a more endotype-based view of severe asthma and suggests a path toward more biologically precise patient stratification.
- Liu S, Lin Z, Zhou J, Yang X, You L, Yang Q, et al. Distinct Airway Microbiome and Metabolite Profiles in Eosinophilic and Neutrophilic Asthma. J Asthma Allergy. 2025 Jun; Volume 18:1003–22. https://doi.org/10.2147/JAA.S521800.
  - ○ This study integrates microbiome and metabolomic data to show that distinct biochemical pathways underlie eosinophilic and neutrophilic asthma. It adds mechanistic depth to airway endotyping by moving beyond taxonomic differences toward functional metabolic signatures.
- Kuks PJM, Kole TM, Kraft M, Siddiqui S, Fabbri LM, Rabe KF, et al. Neutrophilic inflammation in sputum or blood does not define a clinically distinct asthma phenotype in ATLANTIS. ERJ Open Res. 2025 Jan;11(1):00616–2024. https://doi.org/10.1183/23120541.00616-2024.
  - ○ This analysis challenges the use of neutrophilia as a defining feature of non-T2 asthma by showing weak associations with clinical outcomes. It supports a move away from single-biomarker definitions toward more integrated and biologically grounded phenotyping strategies.
- Corren J, Menzies-Gow A, Chupp G, Israel E, Korn S, Cook B, et al. Efficacy of Tezepelumab in Severe, Uncontrolled Asthma: Pooled Analysis of the PATHWAY and NAVIGATOR Clinical Trials. Am J Respir Crit Care Med. 2023 Jul 1;208(1):13–24. https://doi.org/10.1164/rccm.202210-2005.
  - ○ This pooled analysis is particularly influential because it shows that upstream epithelial alarmin blockade can reduce exacerbations even in biomarker-low subgroups. It provides some of the clearest contemporary evidence that biologic efficacy in asthma need not be restricted to classic eosinophilic disease.
- Cahill K, Bernard G, Niswender K, Peebles S, Bacharier LB. Glucagon-Like Peptide-1 Receptor Agonist in the Treatment of Adult, Obesity-related, Symptomatic Asthma (GATA-3) (GATA-3) [Internet]. 2026 Mar [cited 2026 Mar 15]. Available from: https://clinicaltrials.gov/study/NCT05254314#more-information.
  - ○ This trial reflects a conceptual shift toward treating asthma as a systemic, metabolically influenced disease rather than a purely airway-centered inflammatory disorder. Its focus on obesity-related asthma highlights the growing role of metabolic interventions in T2-low and non-classical phenotypes.
- Celis-Preciado CA, Ben Hamou-Kuijpers E, Ramakrishnan S, Howell I, Wechsler ME, Akuthota P, et al. Applying Precision Medicine to the Heterogeneity of Asthma Attacks. Chest. 2025 Nov 13;S0012-3692(25)05695-8. https://doi.org/10.1016/j.chest.2025.11.013 PubMed PMID: 41241145.
  - ○ This work advances the field by proposing that asthma exacerbations themselves should be biologically phenotyped rather than treated as uniform clinical events. It provides a framework for integrating biomarkers, infection, and environmental triggers into acute care decision-making.

## Data Availability

No datasets were generated or analysed during the current study.
